# Neuronal Mechanisms of Voice Control Are Affected by Implicit Expectancy of Externally Triggered Perturbations in Auditory Feedback

**DOI:** 10.1371/journal.pone.0041216

**Published:** 2012-07-17

**Authors:** Oleg Korzyukov, Lindsey Sattler, Roozbeh Behroozmand, Charles R. Larson

**Affiliations:** Department of Communication Sciences and Disorders, Northwestern University, Evanston, Illinois, United States of America; McMaster University, Canada

## Abstract

Accurate vocal production relies on several factors including sensory feedback and the ability to predict future challenges to the control processes. Repetitive patterns of perturbations in sensory feedback by themselves elicit implicit expectations in the vocal control system regarding the timing, quality and direction of perturbations. In the present study, the predictability of voice pitch-shifted auditory feedback was experimentally manipulated. A block of trials where all pitch-shift stimuli were upward, and therefore predictable was contrasted against an unpredictable block of trials in which the stimulus direction was randomized between upward and downward pitch-shifts. It was found that predictable perturbations in voice auditory feedback led to a reduction in the proportion of compensatory vocal responses, which might be indicative of a reduction in vocal control. The predictable perturbations also led to a reduction in the magnitude of the N1 component of cortical Event Related Potentials (ERP) that was associated with the reflexive compensations to the perturbations. We hypothesize that formation of expectancy in our study is accompanied by involuntary allocation of attentional resources occurring as a result of habituation or learning, that in turn trigger limited and controlled exploration-related motor variability in the vocal control system.

## Introduction

In our everyday environment neuronal mechanisms that incorporate information about future states of the body or the environment appear to be critical for driving cognitive functions and behavior [1], [2]. Anticipation of future events might occur on a variety of timescales ranging from seconds to a year [3]. Since expectations can be formed only after learning about previous events, the neuronal mechanisms underlying expectations to some extent can be theoretically conceptualized within Pavlovian conditioning [4] and reinforcement learning frameworks [5]. For example analogous to Pavlovian conditioning, involuntary learning within the perceptual domain creates expectations about future events without an option to influence them. Essential elements of expectation formation, such as detection and learning of regularity, that describe preceding events might be considered as one of the universal computational algorithms utilized by the neuronal networks for information processing. For example, in audition perception of elementary auditory information such as pitch critically depends on sound periodicity [6]. Perceptual cognizance of more complex sensory inputs such as speech and music are critically linked to the extraction of regular patterns from auditory input. For example, it was recently hypothesized that comprehension of complex linguistic information such as syntactic structure depends on extraction of regular patterns from the speech signal and is mediated by the pre-supplementary motor area – basal ganglia circuit [7].

These fundamental processes can be applied to vocalization control, which relies on both voluntary and involuntary mechanisms. Voluntary control is routinely observed in speech and song. Involuntary mechanisms are important for automatically generating compensatory vocal responses for stabilizing the voice in the face of unplanned perturbations in voice auditory feedback. Both the voluntary and involuntary control mechanisms are engaged in a plethora of interactions with the environment. These interactions incorporate rapid reactions to the environmental challenges that are necessary for the vocal control system to be effective in simulating dynamics of vocalizations and predicting the consequences of motor commands before sensory feedback is available [8].

The mechanisms of these predictions were conceptualized within an internal forward model hypothesis [9], [10] that was supported by various motor control studies (for review see: [11]). The forward model hypothesis suggests that the brain employs a copy of the motor commands acting on muscles (termed efference copy) to predict the sensory consequences of movement and to remove sensory effects of its own actions from sensory perception. It follows that during vocalization, similarly to other types of motor behaviors, speakers must make efference copies of the planned motor output, based on short-term predictions about their voice, and when auditory feedback does not match the predictions, the vocal control system reflexively generates corrective responses. However, auditory feedback by itself can be affected by environmental factors that create expectations in the auditory system. It was shown that processing of repetitive auditory events by itself provokes expectancies in the auditory modality that involves an estimation of acoustical parameters of future auditory events including the time intervals prior to the occurrence of the expected event [12], [13], [14]. We hypothesized that the inability to predict perturbations in voice auditory feedback, because of acoustical variability, may require greater neural resources for voice stability control because of the need to closely monitor the feedback signal in order to detect possible unexpected perturbations. We asked the question whether this allocation of resources would affect the vocal responses to the perturbations and would be reflected in cortical Event-Related Potentials (ERPs).

**Figure 1:**
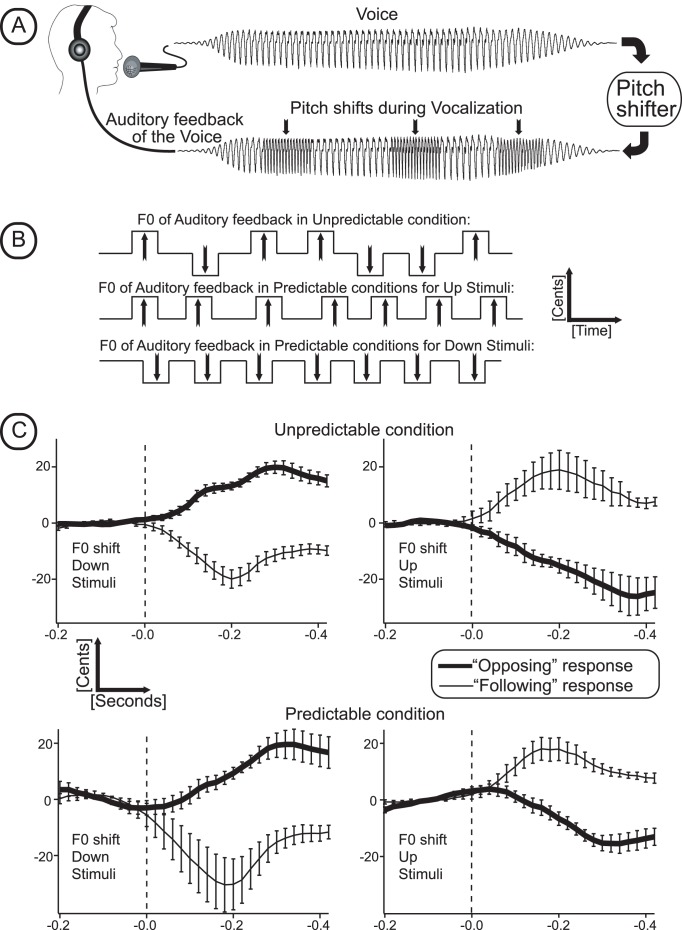
Voice perturbation paradigm and behavioral study results. (A) Schematic illustration of voice output and perturbation stimuli. (B) Schematic representation of experimental conditions showing upward and downward pitch shifts in the unpredictable (top) and predictable conditions (bottom 2 rows). (C) Behavioral (voice) results. Grand-averaged F0 traces across all subjects separately for “Up” and “Down” stimuli with unpredictable and predictable experimental conditions. Thick lines show opposing and thin lines “following” responses. Vertical bars illustrate standard deviations of the averaged F0 traces. Vertical dashed lines represent pitch shift stimuli onset.

To answer these questions, we used a modification of a voice pitch perturbation paradigm (Figure 1A) wherein vocal motor commands are automatically elicited to compensate for pitch-shifted voice auditory feedback [15], [16]. In the present study, repetitive pitch-shift stimuli, which were either predictable or unpredictable in direction (Up vs. Down) were presented to vocalizing subjects (Figure 1B). While most responses to pitch perturbations are compensatory, that is they oppose the stimulus direction and thereby stabilize voice F0, some responses follow the stimulus direction and are therefore destabilizing in nature. The pitch-shift stimuli and the ensuing vocal responses elicited well-documented patterns of ERPs [17], [18], [19], [20]. The patterns of ERP waveforms closely resemble the P50-N1-P2 complex that has been routinely recorded in numerous auditory ERP studies [21]. It is very likely that neuronal generators underlying conventionally recorded auditory ERPs are involved in the generation of the P50-N1-P2 responses elicited by pitch-shifted voice feedback, since the stimulus for the motor responses is a change in frequency of voice auditory feedback. However in contrast to conventional auditory ERP studies, the P50-N1-P2 components in the voice perturbation paradigm are recorded when a subject is vocalizing, i.e. performing a complex, goal oriented motor act. Thus, the dynamic contribution of neuronal activity underling vocal-related motor activity elicited by pitch-shifted voice feedback is likely to affect the ERP components.

The dynamics of this contribution are determined by the complex interactions between auditory and motor components of the voice-control system. These interactions are evident in the studies of neural recordings from the auditory cortex in monkeys, demonstrating that neurons normally suppressed during vocalization become more responsive in the presence of pitch-shifted voice feedback [22]. The suppression of the human auditory cortex during self-vocalization has also been reported in several studies of human brain responses to the onset of voice [23], [24], [25], suggesting that the auditory cortex dampens and delays reactions to self-produced and therefore “expected” sounds. This general dampening hypothesis was supported by ERP studies demonstrating attenuation of auditory cortical responses at a latency of about 100 ms from vocalization onset [24], [26]. In terms of the internal forward model of motor control, these findings were interpreted to mean that an efference copy (or corollary discharge) of the intended vocal output suppresses neural activity related to auditory processing of a subject’s own vocal feedback signal. If the feedback is altered such that it does not match the intended vocal output, the ERP suppression is reduced, and might be entirely eliminated if the voice is recognized as externally generated [27].

The interactions between auditory and motor components of the voice-control system fit nicely within the framework of a hierarchical model of frontal lobe organization and processing [28] and a large scale model of Global Workspace theory [29], suggesting that cognitive processes are built on multiple perception-action cycles. These theoretical accounts have suggested that information circulates between sensory and motor areas through a series of hierarchically organized neural areas and connections that constitute the perception-action cycles according to the temporal structure and regularities in which events occur [30]. The primary motor and premotor areas constitute the lowest levels of that hierarchy and are integrated into automatic well-rehearsed actions that can be observed in the voice control system as a pitch-shift reflex. More complex behavior involving working or implicit memory, leads to switches in attention guided by temporally remote stimuli and utilizes integration of information over longer time intervals requiring integration at higher cortical levels of both perceptual and executive hierarchies [30].

Our methodological approach allowed us to evaluate perceptual and executive hierarchies at both lower and higher levels of processing. While passive auditory perception study designs are useful for experimental manipulations in which expectancy is formed by repetitive auditory events, they cannot evaluate how perceptual expectation might affect motor-related processes since behavioral reactions in purely auditory perception studies are not required. If an auditory perception study involves a voluntary response (for example a button press), all of the neural circuitry elicited by the conscious choice of actions needs to be taken into account to interpret the results. Our study design overcomes some of these limitations because the responses are involuntary. Perception of each expected or unexpected auditory event elicits involuntary behavioral (vocal) and bio-electrical responses that can be used as a quantitative and qualitative measure of the perceptual and efferent components of the cognitive cycle without conscious interference. Thus our study design provides considerable advantages over purely perceptual studies or studies involving perception and voluntary motor responses. Removal of the conscious choice of action greatly simplifies study of the neural circuitry in perceptual-motor cognitive cycles and enabled us to explore how expectancy of sensory inputs can impact the execution of reflexive movements that is used during voice and speech production.

## Methods

### Participants and Procedure

Eleven speakers of American English (7 females and 4 males, mean age: 20.8 years Std. Dev.: 2.5 years) participated in the study. All subjects passed a bilateral pure-tone hearing screening test at 20 dB SPL (octave frequencies between 250–8000 Hz) and reported no history of neurological disorders. All study procedures including recruitment, data acquisition, and informed consent were approved by the Northwestern University institutional review board, and subjects were monetarily compensated for their participation. Written informed consent was received from all participants.

During the test, subjects were seated in a sound-attenuated room and were instructed to vocalize and sustain the vowel/a/for approximately 4–5 seconds at their conversational pitch and loudness levels whenever they felt comfortable, i.e., without a cue. They were informed that their voice would be played back to them during their vocalizations, and they were asked to ignore pitch-shifts in the feedback of their voice. While they were vocalizing, subjects watched a silent movie of their choice and typically paused for 2–3 sec between vocalizations to take a breath. During each vocalization, three pitch-shift stimuli (200 ms duration) with an inter-stimulus interval of 600–800 ms were presented. The first stimulus in each vocalization occurred 500–1000 ms after voice onset.

There were three conditions, comprised of three separate blocks of trials in this experiment. In the predictable-Up condition, all pitch-shift stimuli were in the upward direction (+200 cents), in the predictable-Down condition all stimuli were downward (-200 cents) and in the random condition (unpredictable), stimulus directions were randomly varied from trial to trial (+ or −200 cents). For the predictable Up and Down stimulus direction blocks, subjects vocalized 50 times with 3 pitch-shift stimuli per trial, and therefore received 150 pitch-shift stimuli in each block. In the random stimulus direction block, subjects vocalized 100 times, receiving 3 pitch shifts per trial, resulting in 150 pitch-shift stimuli in each direction. Response magnitudes measured in cents (Cents = 1200×Log2(F2/F1)) were measured from the pre-stimulus and post-stimulus pitch frequencies. The pitch-shift unit, “cents”, is a logarithmic value related to the 12-tone musical scale, where 100 cents equals one semitone. The rise time of the pitch shift was 10–15 ms.

Subjects’ voices were recorded with an AKG boomset microphone (model C420), amplified with a Mackie mixer (model 1202-VLZ3), and pitch-shifted through an Eventide Eclipse Harmonizer. The time delay from vocal onset, the duration, direction, and magnitude of pitch shifts were controlled by MIDI software (Max/MSP v.5.0 Cycling 74). Voice and auditory feedback were sampled at 10 kHz using PowerLab A/D Converter (Model ML880, AD Instruments) and recorded onto a laboratory computer utilizing Chart software (AD Instruments). Subjects maintained their conversational F0 levels and voice loudness of about 70–75 dB, and the feedback signal (i.e., the subject’s pitch-shifted voice) was delivered through Etymotic earphones (model ER1-14A) at about 80–85 dB. The 10 dB gain between voice and feedback channels (controlled by a Crown amplifier D75) was used to partially mask air-born and bone-conducted voice feedback.

### Electroencephalogram

The electroencephalogram (EEG) signals were recorded from 64 sites on the subject’s scalp using an Ag-AgCl electrode cap (EasyCap GmbH, Germany) in accordance with the extended international 10–20 system [31], including left and right mastoids. Recordings were made using the average reference montage in which outputs of all of the amplifiers are averaged, and this averaged signal was used as the common reference for each channel. Scalp-recorded brain potentials were low-pass filtered with a 400-Hz cut-off frequency (anti-aliasing filter), digitized at 2 kHz, and recorded using a BrainVision QuickAmp amplifier (Brain Products GmbH, Germany). Electrode impedances were kept below 5 kΩ for all channels. The electro-oculogram (EOG) signals were recorded using two pairs of bipolar electrodes placed above and below the right eye to monitor vertical eye movements and at the canthus of each eye to monitor horizontal eye movements.

### Behavioral Data Analysis

The voice and feedback signals were processed in Pratt [32], which generated pulses corresponding to each cycle of the glottal waveform, and from this, the F0 of the voice signal (F0 contour) was calculated. These signals along with TTL pulses corresponding to the stimulus onset and direction were then processed in IGOR PRO (Wavemetrics, Lake Oswego, OR). For this processing, the direction of responses in each individual trial was first determined by subtracting the mean amplitude of the voice F0 contour in a 50 ms-long pre-stimulus window (−50 to 0 ms) from a post-stimulus window (50 to 250 ms). Upward and downward responses were defined by positive and negative values, respectively. Averaged responses were then calculated separately for upward and downward responses for each condition. That is, for upward stimuli, an average of the opposing responses (downward) was calculated separately from the “following” responses (upward), and the same was done for downward stimuli. The total number of trials comprising each average (i.e., the number of opposing or “following” responses) for each stimulus direction was also obtained. From the total number of opposing and “following” trials, the percentage of opposing responses was calculated and submitted to statistical testing. A paired t-test was used to test overall significance of the percentage of opposing responses in the random (unpredicted condition) vs. non-random (predicted) conditions. The percentage of opposing responses was also tested in a 2×2 repeated-measures ANOVA (SPSS 19) for response direction (Up or Down), and randomization (random vs. non-random). Follow-up paired t-tests were run on significant differences.

### ERP Data Analysis

The recorded EEG signals were filtered offline using a band-pass filter with cut-off frequencies set to 1.0 Hz and 55.0 Hz (48 dB/oct) and then segmented into epochs ranging from 100 ms before and 500 ms after the onset of the pitch shift. Epochs with EEG or EOG amplitudes exceeding 100 µV were removed from data analysis. At least 100 epochs were averaged for ERP calculation for all subjects. Separate analyses were done for predictable-up and predictable-down stimulus directions, and a difference wave was calculated for each subject and electrode site, by subtracting the ERP to the predictable stimuli from the ERP to the unpredictable stimuli. Then the ERPs and difference waves for each electrode site were grand-averaged across the 11 subjects separately for all experimental conditions and blocks. Based on visual inspection of the scalp distribution of the grand-average data sets, the electrode positions and the latency of the most prominent peaks of the ERPs and difference waves were identified. After that the mean amplitudes across the time windows (length: 20 ms for the ERP and 30 ms for the difference wave) centered over the identified peaks were extracted and submitted for statistical evaluation. For the evaluation of scalp distribution of brain responses and difference waveforms, the measured mean amplitudes were subjected to the voltage normalization procedure [33]. This normalization was done separately for each individual subject’s data subset by dividing the measured mean amplitude at each electrode of the selected array by the square root of the sum of the squared amplitudes of all electrodes in this array.

## Results

### Behavioral Data

All subjects produced a mix of opposing and “following” responses under each experimental condition. Previous studies [15], [34] averaged together the “opposing” and “following” trials of each subject under each condition. However, with the pre-sorting averaging technique used in the present study, in which the opposing and “following” trials were separated before averaging, 55% of responses were opposing and 45% were in the “following” direction (t = 2.70, df 43, p<0.01). The grand averaged responses across all subjects in Figure 1C illustrate the opposing and “following” averaged responses independently. Results of the RM-ANOVA failed to show a significant difference in the percent of opposing responses as a function of response direction (Up vs. Down) or stimulus predictability. There was a significant interaction between response type and predictability (F(1,10) = 6.43, p<0.03). Follow-up paired t-tests showed there to be significantly (t = 2.99, p<0.02) more downward opposing responses in the unpredictable condition (mean 61%) compared to the predictable conditions (mean = 51%). The percentage of upward opposing responses in the unpredictable (mean = 58%) and predictable (mean = 49%) conditions did not differ significantly (t = 1.79, p<0.10).

### ERP Responses

**Figure 2:**
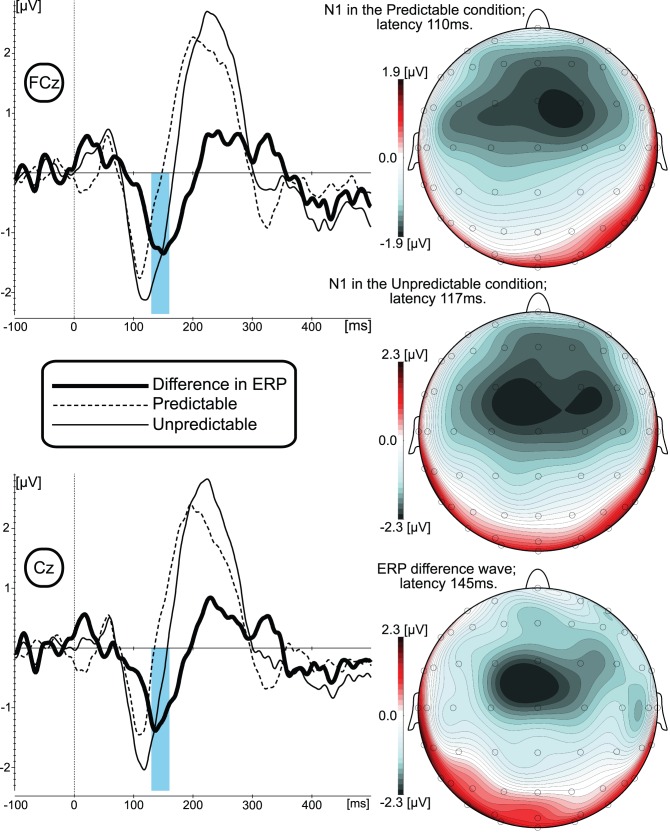
Grand-averaged ERP waveforms and ERP scalp distribution results elicited by the Up shift of the F0. Left panel represents ERPs elicited in predictable and unpredictable experimental conditions measured at two electrode sites that were submitted for statistical analyses. Blue vertical bar depicts time window at which the mean amplitude of the ERP difference wave was measured for the statistical analyses. Right panel represents scalp distribution of N1 response in predictable (top) and unpredictable (middle) experimental conditions as well as the scalp distribution of the ERP difference wave (bottom).

**Figure 3:**
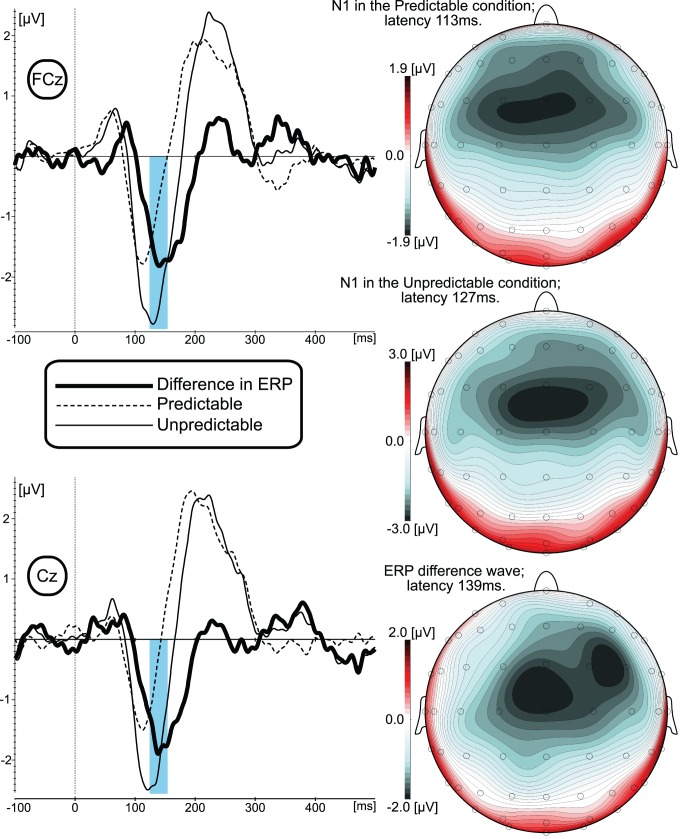
Grand-averaged ERP waveforms and ERP scalp distribution results elicited by the Down shift of the F0. Left panel represents ERPs elicited in predictable and unpredictable experimental conditions measured a two electrode sites that were submitted for statistical analyses. Blue vertical bar depicted time window at which the mean amplitude of the ERP difference wave was measured for the statistical analyses. Right panel represents scalp distribution of N1 response in predictable (top) and unpredictable (middle) experimental conditions as well as the scalp distribution of the ERP difference wave (bottom).

As can be seen from the grand-averaged ERP data (Figure 2 and 3; left panel), the pattern of the P50-N1-P2 ERP responses for both predicted and unpredicted experimental conditions and across Up and Down stimuli are similar to results reported in conventional auditory ERP studies [21]. The most prominent difference between the experimental conditions (calculated by subtracting the ERP waveform to the predictable from the ERP to the unpredictable stimuli) is depicted as a “difference wave” that was maximal within the time range of the N1 response peak, around 145 ms for the Up and around 139 ms for the Down stimulus directions.

### Scalp Distribution Analyses

Visual inspection (Figure 2 and 3; right panel) of the scalp distribution showed that the amplitude of the N1 response and the difference wave were maximal at the fronto-central midline electrodes. In order to statistically validate that these responses were maximal at fronto-central midline electrodes and evaluate the possible differences in scalp distributions between N1 and the expectation-related differences in brain responses, the array of 25 electrodes centered over fronto-central midline electrodes (where N1 and difference waves were maximal) was selected for the scalp distribution analyses. The normalized N1 amplitudes measured between 101–121 ms (predictable condition) and 107–127 ms (unpredictable condition) for Up stimuli and between 103–123 ms (predictable condition) and 117–137 ms (unpredictable condition) for Down stimuli) were compared with 3-way ANOVAs (General Linear Model; Repeated measures) including the following factors: Predictability (predicted vs. unpredicted condition); Frontality (Frontal electrodes F3, F1, Fz, F2, F4 vs. Fronto-central FC3, FC1, FCz, FC2, FC4 vs. Central C3, C1, Cz, C2, C4 vs. Centro-parietal CP3, CP1, CPz, CP2, CP4, and vs. Parietal P3, P1, Pz, P2, P4); Laterality (left F3, FC3, C3, CP3, P3 vs. left-central F1, FC1, C1, CP1, P1 vs. central-midline Fz, FCz, Cz, CPz, Pz vs. right-central F2, FC2, C2, CP2, P2 vs. right F4, FC4, C4, CP4, P4).

ANOVA results for N1 for the Up direction stimuli revealed a significant F(4,40) = 10.328, p<0001 main effect of Frontality. The subsequent Scheffe and Bonferroni Post Hoc tests revealed that this effect was due to more negative mean N1 amplitude at Frontal (−191 µV; p<001), Fronto-central (−224 µV; p<001), and Central (−2 µV; p<001) as compared to parietal (−026 µV) electrodes. For the Down direction stimuli, the main effect of Frontality also was significant F(4,40) = 13.187, p<0001. The subsequent Post Hoc tests revealed that this effect was due to a significant amplitude difference between Frontal (−172 µV; p<001), Fronto-central (−223 µV; p<001), Central (−195 µV; p<001) and parietal (−023 µV) electrodes. The mean N1 amplitude for Down direction stimuli also was significantly (p<034) more negative at the Fronto-central electrodes (−223 µV) as compared with Centro-parietal (−091 µV) electrodes.

The same statistical approach was applied for the evaluation of the difference wave scalp distribution (Figure 2 and 3). In the ANOVA design described above, the predictability factor was substituted for the shift direction factor, Up vs. Down stimuli. ANOVA results revealed a significant F(4,40) = 6.045, p<001 main effect of Frontality and Laterality F(4,40) = 3.271, p<021. The subsequent post hoc tests revealed that the measured amplitude of the difference wave was significantly more negative at Frontal −136 µV; p<042), Fronto-central (−171 µV; p<006), Central (−168 µV; p<001) as compared to Parietal (−001 µV) electrodes. Post hoc tests of Laterality showed that while left-central (−119 µV); central-midline (−143 µV); right-central (−131 µV); right electrodes (−103 µV) were not statistically different from each other, the mean amplitude of the difference wave at the left electrodes (−069 µV) was significantly (p<042) less than at the central-midline electrodes.

### Expectation Effects

Since the results of visual inspection and scalp distribution analyses suggested fronto-central electrodes as sites where responses were most prominent, non-normalized data from 6 fronto-central electrodes were submitted to 3-way ANOVAs (General Linear Model; Repeated measures) including the following factors: Predictability (predicted vs. unpredicted condition); Frontality (Fronto-central FC1, FCz, FC2 vs. Central C1, Cz, C2 vs.); Laterality (left-central FC1, C1 vs. central-midline FCz, Cz vs. right-central FC2, C2). ANOVA results for N1 for the Up direction stimuli revealed a significant F(2,20) = 6.324, p<008 interaction between Predictability, Frontality, and Laterality. The subsequent Scheffe and Bonferroni Post Hoc tests revealed that this effect was due to significantly (p<01) more negative mean N1 amplitudes in the unpredictable condition as compared to the predictable condition for all 6 electrodes. ANOVA results for N1 for the Down direction stimuli revealed a significant F(1,10) = 14.522, p<004 main effect of Predictability (−2.44 µV for unpredicted; −1.52 µV).

The mean amplitude of the difference wave at the FCz and Cz electrodes (measured across a 130- to 160-ms time window for Up and at 124- to 154-msec for Down stimuli) was submitted for the statistical validation of a nonzero value. For the Up stimuli, statistical comparison between measured mean amplitudes of the difference wave and the mean amplitude across the pre-shift interval (duration 100 ms; after baseline correction mean was set to zero) showed that even with the most conservative (nonparametric; very few assumptions) Sign Test statistical approach, the amplitude of the difference wave at Cz was significantly different from zero (Mean amplitude: −1.229 µV; Std. Dev.: 1.532; p<016). At the FCz, the Sign Test comparison did not reach statistical significance (p<071; Mean amplitude: −1.2682 µV; Std. Dev.: 1.394). For the Down stimuli, the Sign Test statistical also confirmed that mean amplitude of the difference wave at Cz (−1.4725 µV; Std. Dev.: 1.054) significantly (p<016) different from zero. At the FCz mean amplitude (−1.3998 µV; Std. Dev.:1.027) was not different from zero (p<071).

## Discussion

Results of the present study demonstrate that repetitive presentation of identical pitch-shifted voice auditory feedback stimuli leads to changes in the vocal control system that can be measured at the behavioral and neural levels. Such stimulation led to a greater percentage of “following” vocal responses and smaller amplitude N1 ERP responses. In contrast, unpredictable stimuli led to more opposing responses and a larger N1 ERP. Since it has been suggested that opposing responses stabilize voice F0 and following responses are destabilizing [34], the findings might suggest a reduction in neural resources that are allocated to the processing of predictable stimuli with a consequent reduction in vocal control. Accordingly, greater neural resources are allocated to unpredictable than to predictable stimuli. In the present study a weakening of F0 stability control caused by the predictable stimuli led to a reduction of N1 amplitude and a significant increase in the number of “following” vocal responses.

From the motor control perspective the increased percentage of “following” responses in the predictable condition can be viewed as an increase in trial-to trial variability in the motor control of F0 stability during vocalization. The significance of variability in motor control was conceptualized within an optimal feedback control framework [35]. This computational-level theory suggests that external perturbations are closely related to the pattern of sensorimotor variability, and optimal motor performance is achieved by exploiting motor variability in redundant (task-irrelevant) subspace or dimensions [35]. In terms of this theory “following” responses in our study can be viewed as a variation of a response into task-irrelevant, redundant dimensions. According to the theory [35], this variation (or variability) is needed for more optimal use of feedback to correct only those deviations that interfere with task goals [35]. Although variability in motor control of F0 stability might originate from several neuronal processes, one of the more plausible sources for the behavioral variability is the motor cortex. The study of variability in firing rate of neurons in the monkey’s motor cortices during visuomotor adaptation suggested that limited and controlled variability represents a motor learning mechanism in which the system can balance the need for stable neuronal configurations of the motor state and neuronal plasticity that enables adaptation and learning [36]. We can hypothesize that similar limited and controlled variability in the motor cortex controlling stability of F0 during preparation for predictable pitch-shift stimuli was the reason for the increase in the percentage of “following” responses.

In light of the forward internal model hypotheses [9], [10], [11], the observation of a decrease in the N1 ERP magnitude and the increased percentage of “following” responses in the predictable condition suggest that implicit expectations about sensory input might modulate the efference copy mechanisms of vocal control. Thus, we may speculate that the effect of predictability of sensory feedback is a factor that mediates the allocation of neural resources involved in the negative feedback vocal control system.

At the level of neuronal generators, the expectation-related differences in ERP responses found in the present study might be interpreted in relation to the biological significance of the N1 response. Results of our scalp distribution analysis showed that although the peak latency of the expectation-related ERP difference wave appear to be a bit later than the N1 response, they both exhibit very similar frontal-central scalp distributions suggesting that they may partially reflect activity of the same neuronal populations. Some auditory perception studies suggested that the auditory N1 ERP component might reflect assignment of pitch or phonemic quality to auditory objects in higher auditory centers [37]. Other auditory perception studies suggested that some neuronal activity underlying the auditory N1 response might be associated with object discrimination [38], percept formation [39] and auditory memory [40], [41]. Study of the auditory N1 response that was elicited by the perception of self- and externally initiated sounds showed that differences in amplitude of the auditory N1 response also reflect discrimination between self and externally initiated sounds, implying involvement of some of the neuronal generators of the auditory N1 in internal prediction mechanisms [42], [43].

Assuming that the N1 measured in auditory perceptual studies is similar to the N1 elicited in the voice perturbation paradigm, we can hypothesize that in addition to processing efference copy based expectations, some of the neuronal generators underlying N1 measured in our study might be associated with higher cognitive aspects of vocal output monitoring. For instance, one of these aspects might be monitoring the results of vocal compensation that involves processing of information about the extracted regularity in the directions of the externally produced voice feedback pitch-shifts during several previous acts of vocalization. This monitoring requires integration of information over longer time intervals and ascribes neuronal activity underlying the expectation-related ERP difference to the higher level of both perceptual and executive hierarchies within the framework of a hierarchical model of frontal lobe organization and processing [28], [30].

Analysis of the timing of the expectation-related ERP differences in the present study provides some support for this hypothesis. In the present study, initiation of compensatory F0 responses occurred before the expectation-related ERP difference reached its maximum value. Previous studies have shown that the latencies of the F0 compensatory responses can be as short as 100 ms [44]. Since the laryngeal muscle contraction times preceding a change in voice F0 are about 30 ms [20], [45], [46], and the latency from transcranial magnetic stimulation of the laryngeal motor cortex to a laryngeal EMG responses is about 12 ms [47], [48], it can be assumed that the earliest the laryngeal cortex could be activated would be about 58 (100−(30+12)) ms following a pitch-shift stimulus. The peak latency of the expectation-related difference in the brain activity in our study was around 135–150 ms, which is too late to be ascribed to the error detection or even motor reaction to the violation of expectation. We suggest that the expectation-related ERP differences might be associated with the N1 generators that are responsible for more advanced sequential stages of environmental scene processing, namely for learning about the regularity of the direction of the shift in the preceding voice auditory feedback.

Current density studies suggest that in parallel to the auditory cortex contributions to the auditory N1 response, additional frontal currents originating from the motor cortex or the supplementary motor area might contribute to this response [49]. Giard et al. [49] hypothesized that these separate, simultaneously active temporal and frontal neural systems could be associated with auditory-motor linkages that short-circuit the highest levels of the auditory system [49]. In light of this hypothesis, the decrement of N1 found in the present study might be interpreted as an allocation of resources away from higher levels of the integrated auditory-motor system controlling stability of F0.

Encoding and remembering this higher order information can be described within the Pavlovian conditioning framework as the process of learning about regularities in the environment. Neuronal mechanisms of this learning are associated with a decline of response magnitude as a function of stimulus repetition, which is termed habituation. Conventionally, habituation is defined as behavioral response decrements that represent “the simplest form of learning” [50]. Habituation is an important aspect of behavioral plasticity [51] and according to a theory proposed by Sokolov [52], reflects the establishment of internal representations (or “neuronal model”) of a stimulus following repeated exposure. It is believed that habituation might allow an organism to filter out irrelevant stimuli and focus on important stimuli. Habituation, as a central feature of stimulus orienting theories is thought to reflect reduced passive attentional processing of a stimulus as its novelty wanes [53]. From this perspective, the decrement of N1 in the present study can be considered as a metric of involuntary attentive mechanisms for vocal stability control when the brain creates moment-to-moment expectancies. The link between attention and moment-to-moment expectancies recently was conceptualized [54], and it was postulated that selective attention is involved in the generation of expectancies of sensory input, and with learning, this mechanism allows behavior to shift from reactive to predictive or a so-called future-oriented brain state [54]. According to this theory, voluntary attention, along with learning, reduces performance variability. Since attention-related brain resources can be easily allocated involuntarily, it is very likely that these same types of resourses were allocated away from the phonation control in the predictable situations of our study and led to increases in variability of F0 stability control. Following this line of reasoning we can hypothesize that formation of expectancy in our study is accompanied by involuntary allocation of attentional recourses occurring as a result of habituation or learning, that in turn trigger limited and controlled exploration-related variability in the F0 control system.

For the understanding of how expectancy can modulate vocal control, it should be noted that studies of implicit sequence-learning suggest that complex sequences of events can be learned only if associations between successive stimuli (perceptual learning) and between successive motor responses are concurrently formed [55]. As applied to our study, formation of expectancy involved concurrently (implicitly) learned sequences of auditory feedback of vocalizations and motor vocal control actions. The question of how the selective attention, learning and expectation-related variables vary in relation to general auditory and motor variables related to the vocal stability control system remains open for future studies.
